# Automatic Body Segment and Side Recognition of an Inertial Measurement Unit Sensor during Gait

**DOI:** 10.3390/s23073587

**Published:** 2023-03-29

**Authors:** Mina Baniasad, Robin Martin, Xavier Crevoisier, Claude Pichonnaz, Fabio Becce, Kamiar Aminian

**Affiliations:** 1 Laboratory of Movement Analysis and Measurement, Ecole Polytechnique Fédérale de Lausanne (EPFL), 1015 Lausanne, Switzerland; 2 Department of Orthopaedic Surgery and Traumatology, Lausanne University Hospital, University of Lausanne, 1011 Lausanne, Switzerland; 3 Department of Physiotherapy, School of Health Sciences HESAV, HES-SO University of Applied Sciences and Arts Western Switzerland, 1011 Lausanne, Switzerland; 4 Department of Diagnostic and Interventional Radiology, Lausanne University Hospital, University of Lausanne, 1011 Lausanne, Switzerland

**Keywords:** sensor location, wearable sensor, IMU-2-segment pairing, I2S pairing, stride-time estimation, side identification, IMU sensor placement

## Abstract

Inertial measurement unit (IMU) sensors are widely used for motion analysis in sports and rehabilitation. The attachment of IMU sensors to predefined body segments and sides (left/right) is complex, time-consuming, and error-prone. Methods for solving the IMU-2-segment (I2S) pairing work properly only for a limited range of gait speeds or require a similar sensor configuration. Our goal was to propose an algorithm that works over a wide range of gait speeds with different sensor configurations while being robust to footwear type and generalizable to pathologic gait patterns. Eight IMU sensors were attached to both feet, shanks, thighs, sacrum, and trunk, and 12 healthy subjects (training dataset) and 22 patients (test dataset) with medial compartment knee osteoarthritis walked at different speeds with/without insole. First, the mean stride time was estimated and IMU signals were scaled. Using a decision tree, the body segment was recognized, followed by the side of the lower limb sensor. The accuracy and precision of the whole algorithm were 99.7% and 99.0%, respectively, for gait speeds ranging from 0.5 to 2.2 m/s. In conclusion, the proposed algorithm was robust to gait speed and footwear type and can be widely used for different sensor configurations.

## 1. Introduction

Thanks to technological advances, inertial measurement units (IMUs) are available in small sizes, at low cost, and are widely used for biomedical applications such as gait analysis [1], rehabilitation [2], sports [3,4,5], injury prevention [6,7], and activity monitoring [8]. In general, the data from single or multiple IMUs placed on body segments are fused to obtain spatiotemporal parameters [9] or joint orientation [10] during movement. While the setup time and ease of preparation are crucial for the popularity of the wearable system, the user must often be very careful to place sensors correctly on each body segment, as the motion analysis algorithms usually rely on sensor configuration. Simple and quick installation/uninstallation of sensors has been considered the highest desired characteristic for practical use of IMU in clinics [11]. We can consider two types of sensor misplacement. The first assumes that the sensor is placed on the correct segment but its orientation with respect to the underlying bone is arbitrary. In this case, the placement error is usually minimized by a functional or anatomical calibration that aligns the sensors with the anatomical framework of the segment [12]. The second type of misplacement which is the subject of this study, is where the sensor is not placed on the correct segment, for example, when switching between the left and right limb or between the lower and upper limb. While a verification procedure could reduce this error, it adds additional load to the user who may not have the technical knowledge. Moreover, it extends the installation time which should be limited, especially when it comes to measurements on patients. In addition to this, any user is prone to human error, especially in multi-sensor applications where each sensor has to be attached to a specific body segment. The error is even more important for applications where the installation of the sensors should be carried out by the patient or his/her entourage. This study investigated solutions for this second type of error, where an automatic pairing of the IMU to the segment (I2S) was proposed to overcome this problem and make the application of IMU motion capture systems simpler.

Several algorithms have been previously proposed for automatic I2S pairing [13,14,15,16,17,18,19,20,21,22,23]. Weenk et al. [22] suggested an algorithm that identified the body segment of the IMU sensors using a decision tree with fixed thresholds, and features were extracted from the amplitude of the IMU signal. The algorithm works appropriately for the same sensor configuration and self-selected range of gait speed in healthy subjects and patients with anterior cruciate ligament injury. However, the amplitude of the IMU signal is highly dependent on the gait speed [24,25], and the performance of the algorithm depends on the walking speed [22]. Mannini et al. [21] developed an accelerometry-based algorithm to recognize the sensor location on the ankle, thigh, hip, arm, and wrist using features in time and frequency domain that compared the amplitude of all sensors together. Although it solved the problem of gait speed, it required having the same sensor configuration, which limits generalizability. Other studies [18,19] developed algorithms to detect the location of smartphones in the breast/hip pocket, bag, or hand during normal walking. Weenk et al. [22] also proposed an algorithm to identify the side by extracting the orientation of the sensors in the global frame and computing the correlation coefficients with the sensor on the sacrum. Another study [25] proposed a method to categorize the side of the feet sensors to side 1 and side 2, but it could not classify to the right and left. Therefore, to the best of our knowledge, there is currently no robust and generalizable method to identify the location and side of IMU sensors on body segments during gait analysis.

The main objective of this study was to develop and validate a biomechanically-driven machine learning algorithm that could accurately identify the location and side of IMU sensors on body segments. To ensure the algorithm’s robustness, we tested it under varying conditions, including different gait speeds, footwear types, and pathological gait patterns. We also designed the algorithm to accommodate a wide range of sensor configurations, whether they involve a single sensor or multiple sensors. In developing the algorithm, we extracted features from individual sensors without making any inter-sensor comparisons. To improve accuracy in a wide range of gait speeds without complicating the I2S pairing, we developed a method to estimate the stride time when the sensor’s location was unknown. We scaled the IMU signals by the estimated stride time to minimize the dependency of the I2S pairing algorithm on gait speed.

## 2. Materials and Methods

### 2.1. Experimental Protocol

**Figure 1:**
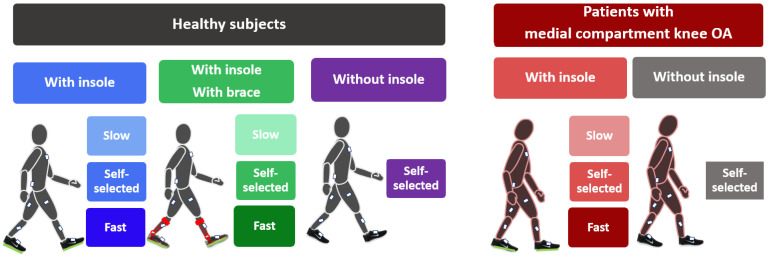
Graphical summary of the experimental protocol, including healthy subjects and patients. All subjects walked at three speed levels: self-selected, slow, and fast while wearing an instrumented insole. All subjects also walked at self-selected speed after removing the insole. To investigate the deviated gait pattern, healthy subjects walked with the brace at three speed levels. OA: osteoarthritis.

A total of 34 participants, including 12 healthy subjects and 22 patients with medial compartment knee osteoarthritis (OA), participated in this study (Table 1). Eight IMU sensors (Physilog 4, GaitUp, Lausanne, Switzerland) were attached to the feet, shanks, thighs, the sacrum, and the trunk and synchronously recorded data at 200 Hz. Each IMU measured the tri-axial angular velocity (${Gyr}_{x}{Gyr}_{y}{Gyr}_{z}$) and acceleration (${Acc}_{x}{Acc}_{y}{Acc}_{z}$). The sensor locations were noted for validation and the sensor orientations with respect to the body segments were arbitrary. Participants were asked to walk along the lab back and forth at three different gait speeds. A pair of instrumented insoles (Pedar, Novel, Munich, Germany) were fixed in the shoes and were used as a reference system to measure the contact time of hind foot with the ground. This insole has been reported as accurate and reliable as force plate in gait measurements [26]. So, we used it as the gold standard for estimation of the stride time. To investigate the robustness of the algorithm to footwear type, extra trials were collected without insoles. To avoid long test duration, these extra trials were performed only at self-selected speed (Figure 1).

In order to simulate abnormal gait patterns and to consider the effect of instruments on gait, healthy subjects repeated the test while wearing an ankle–foot orthosis (Agilium Freestep, Ottobock, Duderstadt, Germany) that decreased the foot progression angle [24] and tibia varus [25]. For each condition (i.e., with/without brace or insole) and each gait speed (slow, self-selected, and fast), six straight walking bouts with minimum two gait cycles were captured. Written informed consent was obtained from all participants, and the study was approved by the local ethics committee (CER-VD protocol 2020-01894).

### 2.2. I2S Pairing

The I2S pairing consists of two parts: automatic segment detection (i.e., trunk, sacrum, thighs, shanks, feet) and then side (left and right) identification for lower limb segments. Prior to analysis, all IMU signals were low-pass filtered (recursive Butterworth 4th order with cut-off frequency at 4 Hz) to remove the noise [27,28].

#### 2.2.1. Automatic Segment Detection

**Figure 2:**
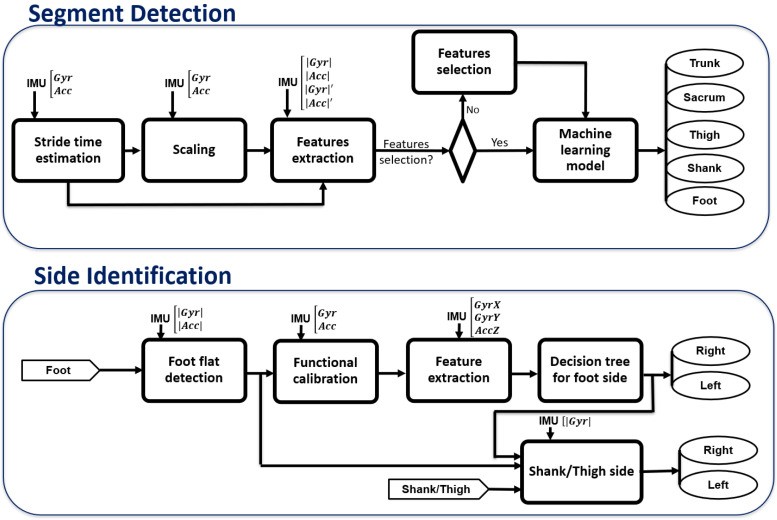
Overview of the I2S pairing with the IMU input corresponding to each of the used signals. The inputs of the side identification algorithm are the foot, shank, and thigh sensors detected in the first part.

We assumed that both the distal location of the sensor and the higher gait speed would increase the amplitude of the IMU signal. This is consistent with a previous study [25] in which the amplitude of IMU signals was higher at distal segments than at proximal and all changed with gait speed. Therefore, to find robust criteria, we first estimated the stride time as a proxy of gait speed and scaled the IMU signals before feature extraction since absolute thresholds typically change with gait speed [22,25] and relative comparisons of features [25] at different locations would limit the application of the algorithm to the similar sensor configuration. In this regard, we first estimated the mean stride time for each walking bout when the sensor location is unknown. Then all IMU signals were scaled by multiplication by the mean stride time to reduce the effect of gait speed and amplify signal difference between segments. The relevant features from |*Gyr*| and |*Acc*| were extracted for a moving window (without overlapping) equal to one mean stride time. The median of the features for different windows in each walking bout was utilized for machine learning (Figure 2).

**Stride-time estimation**—Existing algorithms for estimating gait cycle or stride time rely on knowing the location of the sensor on a specific body segment [9,29,30,31], which is not useful here. We proposed a generic algorithm to estimate the stride time when the sensor location is unknown. In this regard, two analyses were performed on time and frequency domain. In frequency domain, the power spectrum of each IMU signals and norms (i.e., 8 signals: (${Gyr}_{x}{Gyr}_{y}{Gyr}_{z}\left|Gyr\right|{Acc}_{x}{Acc}_{y}{Acc}_{z}\left|Acc\right|$) were computed (FFT function in Matlab 2021a [32]), and the first peak higher than a certain threshold was identified in each of eight spectrums. As initial estimates of the stride time the inverse of the eight multiplicative inverses of dominant frequencies were considered ($Stride{Time}_{FFT}$). Then, we did an estimation in time domain, by assuming that the integration of each angular velocity component (${Gyr}_{x}$, ${Gyr}_{y}$ and ${Gyr}_{z}$) during a gait cycle should be close to zero, because the orientation of the sensor at the beginning and at the end of a gait cycle should be the same. Thus, for each IMU, we found the minimum window size that minimize the root mean square of the residuals of three components of angular velocity. In this regard, the window size (*WS*) was incrementally increased (${WS}^{n+1}={WS}^{1}+\frac{n}{samplingfrequency}$) from the initial value of ${WS}^{1}$ = 300 ms. The initial value of 300 ms was selected to reduce the computation time since the stride time even in fast walking is greater than 300 ms. For each window size, the residual was computed as root mean square of the median of residuals over different not-overlapping windows in each walking bout (Equations (1)–(4)).

(1) $${Res}^{n}={\left({Res}_{x}^{n}\right)}^{2}+{\left({Res}_{y}^{n}\right)}^{2}+{\left({Res}_{z}^{n}\right)}^{2}$$

with

(2) $${Res}_{x}^{n}=median\left(\int_{i}^{i+WS^{n}}{Gyr}_{x}\right)$$

(3) $${Res}_{y}^{n}=median\left(\int_{i}^{i+WS^{n}}{Gyr}_{y}\right)$$

(4) $${Res}_{z}^{n}=median\left(\int_{i}^{i+WS^{n}}{Gyr}_{z}\right)$$

$$for\;i=0:WS^{n}:k\times WS^{n},\;k\in N\;\&\;\left(k\times WS^{n}\right)<signal\;duration$$

To avoid errors sourced from gait initiation and termination (that the residuals are not zero theoretically), the median was used to select the residuals over different windows in each walking bout. The window size associated with the first index *n* that minimizes the residual in Equation (1) was considered as the second estimate of the stride time as shown in Equations (5) and (6):

(5) $$Stride{Time}_{ResidualAnalysis}={WS}^{\hat{n}}$$

(6) $$\hat{n}=\min\left({argmin}_{n}\left({Res}^{n}\right)\right)$$

In the final step, out of eight estimates of stride times based on frequency analysis, the one closest to the second estimate of stride time based on residual analysis was selected as the mean stride time of the walking bout (Equations (7) and (8)).

(7) $$MeanStrideTime=Stride{Time}_{FFT}^{\hat{j}}$$

(8) $$\hat{j}={argmin}_{j}\left(\left|Stride{Time}_{FFT}^{j}-Stride{Time}_{ResidualAnalysis}\right|\right)1\leq j\leq 8$$

This procedure was performed for each IMU sensor. In the case of multiple sensors, the median of the stride times based on different sensors was considered as the stride time.

**Scaling IMU signal by stride time**—The amplitude of the IMU signals is affected by sensor location and walking speed. To minimize the effect of walking speed and have better separation between segments regardless of walking speed, we scaled the IMU signals by multiplying by stride time. This way, the higher amplitude signal in fast walking was reduced via multiplication by the smaller stride time, and the low amplitude signal in slow walking was amplified by larger stride time. We assumed that the scaling should minimize the effect of gait speed on the kinematic profile (e.g., angular velocity, and acceleration) of a single segment while better separating the kinematic differences between segments.

**Feature extraction**—To obtain a more general model regardless of sensor orientation with respect to body segment, for each IMU, the norm (i.e., $\left|Gyr\right|\;and\;\left|Acc\right|$) and derivative of the norm of the gyroscope and accelerometer (i.e., ${\left|Gyr\right|}^{\prime }\;and\;{\left|Acc\right|}^{\prime }$) were used for feature extraction. We extracted the min, max, interquartile, 10th, and 90th percentiles, mean, median, kurtosis, skewness, standard deviation, and mean absolute deviation. We also extracted further features as follow: the percentage of motionless period, the number of peaks and valleys of $\left|Gyr\right|$ and $\left|Acc\right|$, and the number of zero crossing of ${\left|Gyr\right|}^{\prime }$ and ${\left|Acc\right|}^{\prime }$. The motionless period was defined as the time that $\left|Gyr\right|<10\frac{deg}{s}$ and $\left|Acc\right|<1.3\;g$.

Since the window size can affect some features, instead of setting a fixed value, a not-overlapping window size of one stride time was personalized for feature extraction. The median of features extracted from several windows in one walking bout was used for machine learning. Median was used rather than mean to avoid outliers from gait initiation and termination. Since the features were from different sources (gyroscope and accelerometer and their derivatives) with different ranges and units (deg/s, deg/s^2^, m/s^2^, m/s^3^, and unitless), we normalized the features using the z-score method [33].

**Feature selection**—To avoid overfitting, the number of features was reduced by ranking them and selecting an optimum number [34]. The minimum redundancy maximum relevance (MRMR) method was used to rank features based on the importance score [35,36]. Then the number of sorted features was incrementally increased to observe the performance of the trained model on the development dataset. Three criteria were used to assess the performance of the model: misclassification error (MCE), F1-measure (harmonic mean of precision and sensitivity), and area under the roc curve (AUC). The performance graph versus the number of features was considered to select the optimum number of features.

**Machine learning model training**—Healthy participants were randomly divided 75–25% into training and development sets, and patients were considered only for the test set such that all of a participant’s data resided in only one set. Using the selected features, we trained two common machine learning models, including decision tree (gini criterion) and support vector machine (with linear, cubic, and Gaussian kernels) in Matlab 2021a. To select the classifier, we compared their accuracies using repeated cross-validation [37]. The model’s input could be one to eight IMUs attached to one of the body segments, including foot, shank, thigh, sacrum, or trunk.

#### 2.2.2. Side Identification of Lower Limb Segments

Once the segment of each IMU was detected, the side (i.e., right/left) needed to be identified for thigh, shank, and foot. The proposed algorithm starts with right/left segment identification of feet sensors, and then using this information, right/left shank and thigh were recognized.

**Figure 3:**
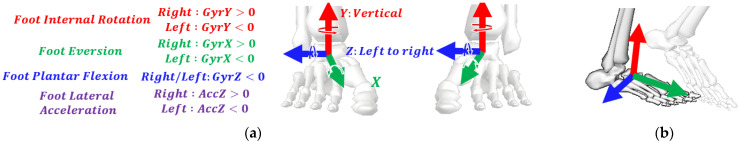
(**a**) Anatomical frame of the foot: with the Y-axis aligned with the gravity in the foot flat period, the Z-axis aligned with the plantar/dorsal flexion with direction from left to right, and the *X*-axis formed by the external cross of the Y and Z. In this frame, the plantar flexion of both feet is reflected as negative signals in *Gyr_z_*. However, the internal rotation and eversion of the right foot led to positive signals in *Gyr_y_* and *Gyr_x_*, respectively, while negative on the left side. (**b**) Foot clears the ground by rotating negatively around the Z-axis in almost all pathologic patterns.

**Foot side**—Assuming that the sensor attachment can be arbitrary, the orientation of the sensor with respect to the foot is then unknown. Aligning the sensor frame to the anatomical (with the Y-axis as vertical, the *X*-axis of walking direction, and the Z-axis from left to right) would lead to an inverse sign of the gyroscope signal during internal rotation and eversion of right and left feet while the similar sign for plantar flexion (Figure 3). Therefore, after aligning the sensor frame with the anatomical foot frame, the internal rotation of the right and left foot would result in positive and negative signs of ${Gyr}_{y}$, respectively. The different signs also occur in eversion in ${Gyr}_{x}$ and lateral acceleration in ${Acc}_{z}$. To benefit from these three discriminant features between right and left, first, the rotations that align the sensor frame with the foot frame are required. This procedure is called functional calibration and was performed in two steps: first, the Y-axis of the foot sensor was aligned with gravity during the foot flat period, and second, rotation was performed around the new Y-axis to align the Z-axis with the mediolateral axis of the foot [38]. We hypothesized that the main movement of the foot during gait is plantar/dorsal flexion that occurs around Z-axis. Therefore, a principal component analysis (PCA) on the gyroscope signals was performed during gait to find the principal axis of the movement [38]. However, the direction of the axis (from left to right or vice versa) was not fixed yet. To confirm the direction, the sign of pitch angular velocity after the foot flat period was used to correct the direction of the Z-axis. We selected the sign of pitch angular velocity of the foot because almost in all normal and pathologic patterns, to clear the foot from the ground, the hind foot leaves the ground sooner than the forefoot, which leads to a negative sign of *Gyr_z_*. The following steps summarize the side identification of the foot sensor.

1. **Foot flat detection**: to approximately detect the period that the foot is flat, find the periods that |*Gyr*| < 5 deg/s for at least 15% of the stride time (in fast walking, the foot flat period can decrease up to 15% of the stride time).
2. **Functional calibration**
  - Rotate the signal to align Y-axis with gravity during foot flat.
  - Find the mediolateral axis of the foot by implementing a PCA on the rotated signal.
  - Rotate the signal around the new Y-axis to align Z-axis with foot mediolateral axis.
  - Check the sign of *Gyr_z_* after the foot flat; if positive, rotate the signals by 180 degrees around Y-axis to have the data in anatomical frame with the Z-axis pointing from left to right for both feet.
3. **Feature extraction**
  - Find the index of the first peak of |*Gyr*| after foot flat.
  - At this index, extract the value of *Gyr_x_*, *Gyr_y_*, and *Acc_z_*.
  - Take the median of these three features for several gait cycles in each walking bout.
4. **Decision tree** for side identification of the foot sensor.

**Shank and thigh side**—The side of the foot was used to determine the side of the shank/thigh. During the foot flat period (identified by the foot sensor), the contralateral shank and thigh are in swing phase with higher amplitude of IMU signals. Therefore, during the foot flat period (right or left does not matter), the average of |*Gyr*| was computed for both shanks/thighs. The sensor with a smaller value was labeled similarly to the associated foot.

### 2.3. Validation

To evaluate the accuracy of stride-time estimation, we used data from the Pedar insole as reference system. To compute the reference stride time, we considered the time difference between two consecutive heel strikes detected when the force reached a threshold equal to 5% of body weight [9]. This threshold was selected based on the previous study [9] to have a similar reference system. The mean (SD) error of the estimated stride time was reported only for the tests with the Pedar system (see Figure 1).

To compute the classification metrics, we used the one-vs.-rest strategy [33] and converted a multiclass problem to a series of binary tasks for each sensor. So, we reported each sensor’s precision, accuracy, sensitivity, specificity, and F1-measure. To report the performance of the whole classifier, we used weighted analysis [39] because we had imbalanced classes for the first part of the algorithm (the sacrum and trunk were half of the feet, shanks, and thighs). In addition to validating the whole algorithm, we evaluated the performance of side identifications of foot and shank/thigh separately because bilateral IMU on feet is a very common sensor configuration. In this regard, the algorithm’s input was only feet sensors for foot side identification, and the output was right and left foot. For shank/thigh side identification, the input was a foot sensor with its side label (right/left) and both right and left shank/thigh sensors, and the output was right shank/thigh and left shank/thigh. To investigate the performance of the algorithm with less than eight sensors, we examined all possible sensor configurations, including single sensor to seven sensors.

## 3. Results

In total, 504 walking bouts (216 with insole, 216 with insole and brace, 72 without insole and without brace) of healthy subjects and 528 walking bouts (396 with, 132 without the insole) of patients with medial compartment knee OA were obtained. Each walking bout included at least two gait cycles and a maximum of ten gait cycles.

### 3.1. Stride-time Estimation

**Figure 4:**
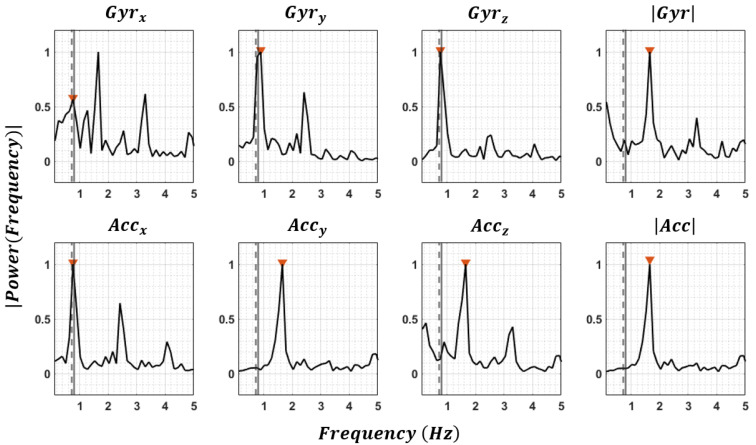
Fast Fourier transform of the gyroscope and accelerometer signals for one walking bout of a healthy subject for the trunk sensor. The first estimates of the stride time corresponding to the first peak in the frequency spectrum with a minimum amplitude of 0.5 are indicated by 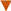. The dotted vertical line shows the second estimate of stride time obtained through Equation (1). The solid vertical line indicates the measured stride time using the instrumented insole. In this case, the first estimates of stride time (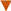) extracted from *Gyr_x_*, *Gyr_z_*, and *Acc_x_* were similar and had a minimum absolute difference with the second estimate (dotted line), so it was considered as the mean stride time of this walking bout.

Figure 4 indicates the frequency spectrum of eight components of the gyroscope and accelerometer signals for one walking trial of a healthy subject where a threshold of 0.5 was selected to identify the first frequency peak. The frequency value obtained through the optimization process (Equation (1)) is illustrated with the vertical dashed line.

The mean ± standard deviation of the error for estimating the averaged stride time compared to reference system (instrumented insole) was 0.00 ± 0.08 s. The error was 0.02 ± 0.18 s when only dominant frequencies from fast Fourier transform were utilized. The best and worst accuracies were for the foot sensor (0.00 ± 0.05 s) and sacrum sensor (−0.06 ± 0.27 s) before taking the median of all sensors.

### 3.2. Impact of Stride Time Scaling

**Figure 5:**
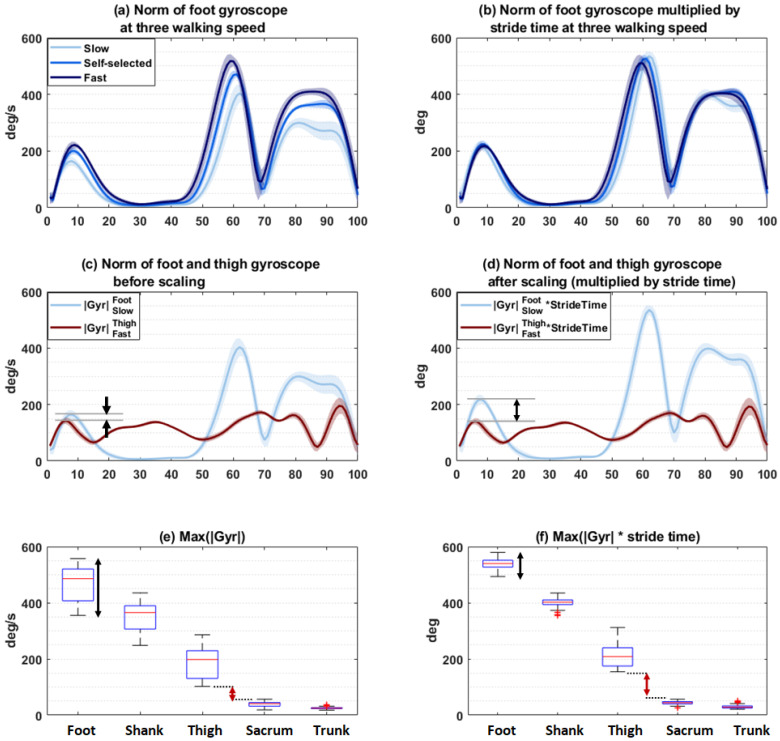
Effect of scaling by stride time. The amplitude of the foot gyroscope signal |*Gyr^Foot^*| at different gait speeds (**a**) before and (**b**) after scaling shows more similarity. Comparison of the amplitude of the |*Gyr^Foot^*| with |*Gyr^Thigh^*| at different gait speeds (**c**) before and (**d**) after scaling showing more separation. Features corresponding to the maximum of |*Gyr*| at different locations (foot, leg, thigh, sacrum, trunk) (**e**) before and (**f**) after scaling, showing less variability at the same location (black arrow) and more separation between locations (red arrow).

The gait speed varied in a wide range between 0.5 and 2.2 m/s. To reduce the effect of gait speed on IMU signals, before feature extraction, *gyr*oscope and accelerometer signals were scaled by multiplying by the mean stride time. After scaling, as shown in Figure 5 for a single sensor (i.e., Figure 5a vs. Figure 5b and Figure 5c vs. Figure 5d), the signals at different speeds became more similar to each other and less dependent on walking speed.

Furthermore, as shown in Figure 5c, the max amplitude of |*Gyr^Foot^*| during stance at slow walking was very similar to the norm of angular velocity of the thigh sensor |*Gyr^Thigh^*| in fast walking, while after scaling, this feature became more discriminant between the sensors (Figure 5d). The boxplot of the features indicated that scaling resulted in more intraclass similarity and higher interclass difference. For example, the maximum of |*Gyr*| could vary in a wide range for the same sensor location and overlap with other sensor locations (Figure 5e), while after scaling (Figure 5f), the range of variation for the same location decreased, and the difference between locations increased.

### 3.3. Segment Detection

**Figure 6:**
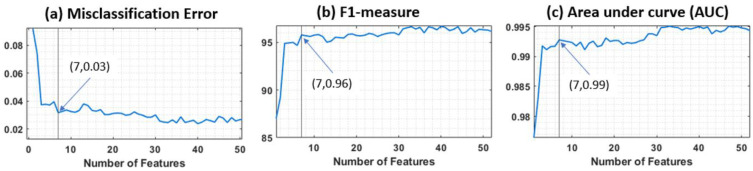
The performance versus the number of features based on the development dataset. (**a**) Misclassification error (MCE), (**b**) F1-measure, and (**c**) area under receiver operating characteristic curve (AUC) of the classifier vs. the number of features giving an optimum number of seven features with MCE = 0.03, F1-measure = 0.96, and AUC = 0.99.

The importance score of the ranked features (Table S1) extracted from MRMR after scaling by stride time and z-score normalization is illustrated in Figure S1. The performance of the segment detection model versus the number of features (ranked based on MRMR) (Figure 6) proposed that seven features were an optimum point based on three evaluation criteria: MCE, F1-measure, and AUC. So, the final model was trained with the first seven features: interquartile range of |*Gyr*|, kurtosis of |*Acc*|’, number of zero-crossing of |*Gyr*|’, minimum of |Acc| and |*Gyr*|, skewness of |*Gyr*|’, and mean of |*Gyr*|’.

There were no significant differences between the accuracies of the decision tree and the support vector machine classifiers, and we selected the decision tree because of its simplicity and interpretability. The weighted precision and sensitivity of the segment detection algorithm were 99.0% and 98.9%, respectively (Table 2a). The performance of the segment detection algorithm depends on the sensor location, with the highest F-measure for the foot (1.00) and the lowest value (0.96) for the sacrum (Table 2a). Specificity and accuracy were above 99%, while trunk and sacrum sensors showed minimum precision (97.5%/94.5%) and sensitivity (94.7%/97.5%). The precision and sensitivity of the whole I2S pairing algorithm, including the segment detection and side identification, were 99.0% and 98.9%, respectively (Table 2d). The misclassified sensors were all sourced from the first part (segment detection). In the case of majority voting of different trials of one subject, the precision enhanced up to 100%. The side identification algorithm perfectly identified the side of the foot with only three features and the side of the shank and thigh with only one feature.

Among all possible sensor configurations (including single sensor to seven sensors) the precision decreased in two configurations; first, a single sacrum sensor (96.2%), and second, two sensors on sacrum and trunk (97.1%). In other configurations the performance was similar or higher than the eight-sensor configuration.

## 4. Discussion

In this study, a novel framework was proposed for the automatic detection of sensor position on the body segment during walking in order to save time and energy for the end user of wearable IMU. First, a new algorithm was designed for an accurate estimation of stride time independent of sensor location. Then, by stride-time scaling and using training data obtained from healthy subjects, the proposed I2S pairing classifier was trained and was able to detect sensor locations in a wide range of walking conditions, including different gait speeds, different footwear (with/without insole), and in patients suffering from medial compartment knee OA. Finally, an algorithm using foot kinematics detected the sensor placement on each side of the lower limbs.

One major specificity of the proposed algorithms is the possibility for I2S pairing when single or multiple sensor configurations are used. The only part of the algorithm that could alter with the number of sensors was stride-time estimation where it used the median of all sensors. The accuracy of the algorithm decreased slightly (98.9% vs. 99.7%) in case of single sensor on sacrum or two sensors on sacrum and trunk due to error in stride-time estimation. Because in sacrum location sometimes the step time can be taken as stride time and the scaling with a smaller value led to a lower-amplitude signal and the classifier would categorize it as the trunk sensor. However, the proposed algorithm does not compare the sensors together (for example, if this sensor is not foot, shank, thigh, trunk, then it should be sacrum). Such an approach is independent of the number of IMUs, meaning that instead of eight sensors if only one sensor is given, the algorithm can still recognize the body segment. This is clinically significant because it expands further its application to a wide variety of sensor configurations. Specifically, the configuration of two sensors on the feet is very common in sports [38], rehabilitation [40], and clinical gait analysis [1] in children and adults, and the algorithm can perfectly identify the side of the foot, even with an only one foot sensor. Moreover, the I2S pairing classifier used features from the norm of the gyroscope and accelerometer, so no caution is required for sensor orientation which drastically facilitates the preparation procedure.

The performance of the proposed I2S pairing algorithm was high (e.g., the overall accuracy of 99.7%) in a wide range of gait speeds from 0.5 to 2.2 m/s, while it is known that the gait speed impacts the profile of the IMU signal [24]. Similar to previous studies [20,22], we noticed that the amplitude of the IMU signals was higher in distal segments, which was very helpful for distinguishing distal from proximal segments. However, this amplitude also depends on the gait speed [22] and interferes with the sensor location’s effect. For example, the amplitude of the thigh sensor in fast walking was very similar to the foot sensor in slow walking. Thus, setting a fixed threshold for the features related to the signal amplitude may not be sufficient when it comes to a wide range of gait speeds. Graurock et al. [25] addressed this issue by relative comparisons between the sensors and acquired a 99.2% successful pairing rate in slow speed and 100% in medium and fast walking. However, relative comparisons or features that require information on more than one sensor require the sensor configuration to be exactly the same as their method. To overcome this limitation, Weenk et al. [22] removed the features extracted from more than one sensor, and the success classification rate decreased from 97.5% to 75.9%. Compared to the existing results, our method performed with a higher success rate of 97.7% in all walking conditions and free sensor configuration. The stride time scaling, as proposed in this study, increased the similarity of the signals within different trials of one subject while contributing to being less sensitive to the range of speed and leading to better discrimination of sensor location. Such as scaling might be a helpful method for further analysis where lowering the impact of speed on the variability of the IMU signals is required.

Another outcome of the proposed study was estimating stride time without knowledge about the sensor location by combining the fundamental frequency detection with an optimization rule in the temporal domain. Such as stride-time estimation could be relevant for applications using smartphone’s IMU where the placement of the smartphone changes during the day (e.g., in the pocket in the thigh, upper or lower trunk area). The mean and standard deviation of the error was 0 ± 80 ms, which was higher than the best existing algorithms, ranging from −9.7 ± 7.5 ms on the dorsal foot to 51.9 ± 47.5 ms on the shank [40]. Nevertheless, after I2S pairing, when the sensor site is identified, in the next layer, the existing algorithms (for the specific sensor locations) for event detection [41] can be used and update the stride time.

The whole algorithm was trained only on 12 healthy volunteers and tested on 22 patients with medial compartment knee OA (who were planned for surgery) walking with/without insole at different speeds. Using a totally new dataset for tests, compared to leave-one-subject-out or k-fold validation, ensures more reliable results. For segment detection, it used only seven features, and for side identification, only three features. A small number of features eliminated the risk of overfitting and extended the generalizability of the algorithm [42]. Furthermore, the robustness of the algorithm in walking with/without insole and brace was confirmed.

Compared to a previous study [22] that requires a minimum of 6 s of walking, this algorithm requires a minimum of only two gait cycles. About 20% of the test dataset included only two gait cycles, and the sensors were classified correctly. So, it requires less data for real-time applications.

This study also has some limitations. The first one was that the other sensor locations, including wrist, arm, and forearm, were not examined while they were used for some applications. The second limitation was the necessity of one foot sensor for side identification of shank/thigh sensor that should be addressed in future studies, for example, by detecting the foot flat using shank and thigh instead of foot [43]. Moreover, we designed the algorithm only for walking, and we did not validate it on other tasks. However, there are developed algorithms that can identify the walking bouts among different physical activities [44] and provide the input for the classifier. Future research could build upon these results by including the upper extremities. The features used for side identification of foot side would be beneficial to identify the side of the wrist IMU as well and can be widely used in smart watches.

The proposed I2S pairing algorithm can decrease the risk of error and facilitate the use of IMU in current clinical applications for health professionals who are not explicitly trained in movement analysis. Furthermore, it opens new opportunities for applications by the patient without external support, including self-rehabilitation, self-measurement in real life, and remote patient monitoring.

## 5. Conclusions

The method proposed here can automatically detect the body segment belonging to the IMU sensors during walking in five common sites, including the foot, shank, thigh, sacrum, and trunk. Its performance was robust in patients with medial compartment knee OA over a wide range of gait speeds (0.5–2.2 m/s) and with different footwear types. The method can be used for many different sensor configurations where the input could be a single or multiple IMU. It can perfectly identify the side of the foot sensor and, subsequently, the side of the shank and thigh sensors. This way, it provides a plug-and-play solution where the user does not need to spend time and effort checking the sensor location, facilitating the use of IMU-based gait analysis systems for non-professionals and decreasing the risk of errors and unusable measurements.

## Figures and Tables

**Table 1:** Participant demographics.

Group	Age	Sex	Height (cm)	Mass (kg)
Healthy subjects (N = 12)	34.3 ± 9.5	11 males 1 female	177.5 ± 6.5	77.3 ± 16.1
Patients (N = 22)	45.4 ± 11.6	16 males 6 females	173.7 ± 10.1	87.6 ± 15.7

**Table 2:** The performance of the decision tree classifier (a) for the segment detection (b) for side identification of feet sensors, (c) for side identification of the shank and thigh, and (d) for the whole I2S pairing algorithm. For (b) and (c), the accuracy is reported based on the correct input of each part, i.e., the input for the side detection of the foot sensor was only feet sensors.

(a) Segment detection classifier					
	**Accuracy**	**Precision**	**Sensitivity**	**Specificity**	**F1-Measure**
Foot	100.0	100.0	100.0	100.0	1.00
Shank	100.0	99.9	100.0	100.0	1.00
Thigh	99.9	100.0	99.7	100.0	0.99
Sacrum	99.0	94.5	97.5	99.2	0.96
Trunk	99.0	97.5	94.7	99.6	0.96
Overall	99.7	99.0	98.9	99.8	0.99
(b) Side identification of foot sensor *					
Right foot	100.0	100.0	100.0	100.0	1.00
Left foot	100.0	100.0	100.0	100.0	1.00
(c) Side identification of shank/thigh based on a labeled foot sensor **					
Right Shank	100.0	100.0	100.0	100.0	1.00
Left Shank	100.0	100.0	100.0	100.0	1.00
Right thigh	100.0	100.0	100.0	100.0	1.00
Left thigh	100.0	100.0	100.0	100.0	1.00
(d) The whole I2S pairing algorithm					
Right foot	100.0	100.0	100.0	100.0	1.00
Left foot	100.0	100.0	100.0	100.0	1.00
Right Shank	100.0	99.8	100.0	100.0	0.99
Left Shank	100.0	100.0	100.0	100.0	1.00
Right thigh	99.9	100.0	99.4	100.0	0.99
Left thigh	100.0	100.0	100.0	100.0	1.00
Sacrum	99.0	94.5	97.5	99.2	0.96
Trunk	99.0	97.5	94.7	99.6	0.96
Overall	99.7	99.0	98.9	99.8	0.99

* The input of the algorithm was foot sensors. ** The input of the algorithm was shank/thigh sensors and one-foot sensor and its side.

## Data Availability

The data presented in this study are available upon request from the corresponding author.

## Supplementary Materials

The following supporting information can be downloaded at: https://www.mdpi.com/article/10.3390/s23073587/s1, Figure S1: Feature importance scores; Table S1: Features ranking.
