# Supplementary material for: Automatic Body Segment and Side Recognition of an Inertial Measurement Unit Sensor during Gait
Source: Sensors (Basel). 2023 Mar 29;23(7):3587. doi: 10.3390/s23073587 (PMC10098809; doi:10.3390/s23073587)
Supplement: Supplementary file 1 [file sensors-23-03587-s001.zip › sensors-2232962-supplementary.pdf]

sensors

MDPI

Supplementary Material

Table S1. Feature's ranking based on importance score computed by maximum relevance and minimum redundancy method.

Rank	Feature	Rank	Feature	Rank	Feature	Rank	Feature
1	IQR(|Gyr|)	14	mad(|Gyr|)	27	NPosPeak(|Acc|)	40	mean(|Acc|)
2	kurtosis(|Acc|')	15	skewness(|Acc|)	28	max(|Gyr|)	41	max(|Gyr|')
3	ZeroCrossing(|Gyr|')	16	median(|Gyr|')	29	P90(|Acc|)	42	P10(|Acc|')
4	min(|Acc|)	17	skewness(|Gyr|)	30	min(|Gyr|')	43	P10(|Acc|)
5	min(|Gyr|)	18	max(|Acc|)	31	MotionLess(|Gyr|)	44	MotionLess(|Acc|)
6	skewness(|Gyr|')	19	skewness(|Acc|')	32	mean(|Gyr|)	45	std(|Acc|)
7	mean(|Gyr|')	20	skewness(|Gyr|')	33	median(|Acc|')	46	IQR(|Acc|)
8	min(|Acc|')	21	P90(|Gyr|')	34	std(|Gyr|')	47	median(|Acc|)
9	NNegPeak (|Gyr|)	22	NPosPeak(|Gyr|)	35	NNegPeak(|Acc|)	48	mad(|Acc|)
10	P90(|Gyr|)	23	std(|Gyr|)	36	median(|Gyr|)	49	mad(|Acc|')
11	kurtosis(|Gyr|)	24	kurtosis(|Acc|)	37	kurtosis(|Gyr|')	50	ZeroCrossing(|Acc|')
12	P10(|Gyr|)	25	IQR(|Gyr|')	38	mad(|Gyr|')	51	IQR(|Acc|')
13	P10(|Gyr|')	26	max(|Acc|')	39	P90(|Acc|')	52	std(|Acc|')

Mad: mean absolute deviation, P10: 10th percentile, P90: 90th percentile, IQR: interquartile range, NNegPeak: number of valleys, NPosPeak: Number of peaks, MotionLess: percentage of the motion less period, ZeroCrossing: number of zero crossing.

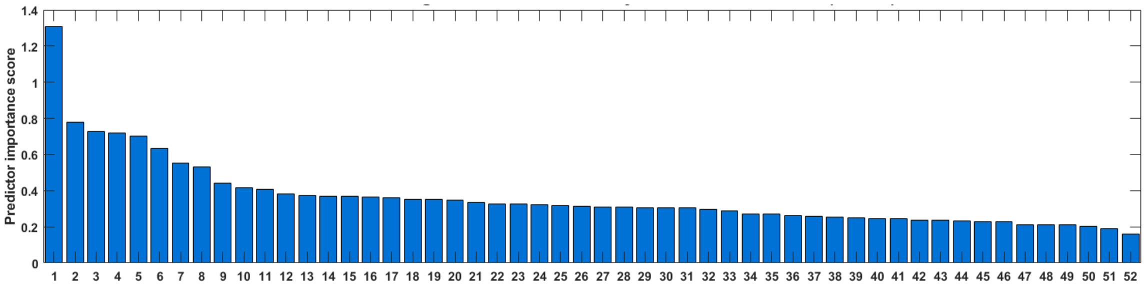

Figure S1. Feature's importance score computed by minimum redundancy maximum relevance method for 52 features.
